# Economics of Philanthropy

**Published:** 1898-05-21

**Authors:** 


### The Institutional Workshop.

### ECONOMICS OF PHILANTHROPY.

### VIII.—REFORMATORIES AND INDUSTRIAL SCHOOLS.

It is not necessary to go out of one's way to prove that crime is an expense as well as a danger to society. Therefore it is not necessary to show that anything that prevents crime is an economical advantage. Besides the ordinary machinery for bringing up the lieges in the paths of honesty and industry, we have special institutions for redeeming those young persons who have lapsed into crime, but whose faults may be due to an unfortunate environment as much as to an innate tendency to evil. Now this innate tendency, which our forefathers would have called original sin, and our less theological and more scientific age terms heredity, forms the crux upon which the question of the economic justification of industrial schools, reformatories, and similar institutions must turn.

We have a number of gloomy-souled philosophers who seem to think that the only way to diminish crime is to hang every creature who has ever been guilty of the smallest peccadillo. They quote the dreary history of the Jukes family, and similar tribes, who from generation to generation have preyed on society. And one cannot deny that in some families the criminal taint seems to be ineradicable. But one might quote in return the history of those aristocratic Australian families whose first known ancestor was a transported sheep-stealer, and who yet seem to be as honest as their neighbours. Or one might bring into court the honoured descendants of those impartial border reivers, who

"Stole the beeves that made their broth,
From England and from Scotland both."

Their descendants are not employed in cattle-lifting to-day. The ideals of the whole civilised world are so changed from what they were a few centuries ago, we are all of us so unlike our grandfathers, that it is only fair to assume that even those who to-day are chiefly notable for their nomadic and predatory instincts may yet leave descendants as far removed from the present bearers of their dishonoured name as an Englishman of our day is from the savages who fought with Cæsar's legions. We want to know, not only the genealogical tree of the Jukeses, but also the circumstances and environment of the original Jukes, before we assume that all the children of pauper and criminal parents are doomed to be inevitably paupers and criminals too.

Now, it cannot be denied that a certain proportion of the pupils at our industrial and reformatory schools do, on leaving them, fall into bad courses. To what extent does this fact justify the contention that they are useless? The point can only be settled by statistics, and these are not always easy to obtain. But it is obvious that the good that these institutions try to do is of two kinds. There is the intention to teach these children, whose home environment is usually bad, some honest means of earning a livelihood; and there is always the desire to remove the children from that environment. While the children are detained in the school this object is secured; but it is difficult to control them afterwards. When the term of detention in the industrial school is over—and, be it remembered, the children there are not criminal, but were sent there sometimes because they were unruly and unmanageable at home, and sometimes because their parents were unsatisfactory—the parents claim their children again. Where do they take them? Back to the old low home, where virtue and honesty are never heard of. Is it the industrial school that is to be blamed if one day these children come up as criminals? Is it not rather the environment to which they returned when their stay at the schools was over? Those who take an interest in these institutions will tell of the way that disreputable parents come to claim their children as soon as the latter are allowed to leave. Some of this eagerness may be due to natural affection; but in the main it arises from a desire to have their children work for them, maintain them, honestly or dishonestly, and they will not be too particular which.

Now, if a young person, on returning to the outer world, is at once restored to a bad environment, there is little cause for wonder if he falls again into bad courses. It is, on the whole, easier to steal than to get work, especially when your friends and relations are better known to the police than to the employers of labour. It is not any failure in industrial schools and reformatories themselves that sends their pupils to gaol; it is the bad environment to which the children return. Placed, if that were possible, in totally new surroundings, honest work made as easy to them as crime is now, it is probable that [illegible] greater number of honest men would emerge from t[illegible]e reformatory than is now the case. It is the actual external influence of the parents, far more than any inheritance of criminal instinct which these parents bequeath, that sends the children back to the paths of evil; and it is to be noted that it is just those parents who were most anxious to get their children taken into the reformatory or industrial school, who swore most fervently that they were unable to control them, who, in short, wanted the expense and responsibility of their offspring taken off their hands when they ought to have maintained them, that most eagerly claim them when, honestly or dishonestly, they are able to be a help to them.

Given a free hand, the reformatory does admirable work. We have at hand more complete statistics of the work accomplished by the reformatory at Elmira, in the State of New York, than of any British institution of similar character. The authorities at Elmira reckon that they succeed in rescuing from evil courses 82 per cent. of the convicts that have been under their charge. It may be admitted that the Elmira convicts are the best of their class. They are young men, between the ages of 16 and 30, who have been convicted of crime for the first time. They are thus old enough to realise both the folly and wrong of sin, and yet not so set in criminal ways as to be beyond hope of reformation. Moreover, they are there taught so many trades that those who have never been able to earn a honest living before can do so when they leave; and others are absolutely able, on going out of the reformatory, to make a better income than they did before. Education, intellectual, technical, and moral, is carefully given them, with reference to their maturity in age and experience, while keeping in mind the comparative poverty of their previous opportunities. And, as has been said, they are of an age to profit by their chances. Younger boys when sent to a reformatory are more conscious of the restraint to which they are subjected; and when at last they are released, they are quite likely to carry their joy in liberty to the point of lawlessness. Reformatory children, and as a rule those brought up under institutional regulations, generally do best when they are placed in some position under strict discipline. They form good recruits for army or navy, not because they are drawn from the criminal or disreputable classes, although we have unfortunately a popular notion that the service of the Queen is the last any man should enter, but because they are accustomed to give unquestioning obedience, while they are comparatively little accustomed to deciding questions of moment for themselves. Going to work where discipline is still strict, and experience of life larger, they obtain just the training they require, and their record is often a most honourable one.

The success of a reformatory lies not only in what is done for the inmates, but in what can be done for them when they go out. Those who conduct them must be in touch with the outside world, and have both the will and the power to place their "graduates" in suitable positions. The same holds good, in even a more marked degree, of prisons and discharged prisoners. The penitence of a criminal, however sincere, can rarely save him from relapse if, on his release, he returns to his old companions and his old surroundings. The work done by prisoners' aid societies is therefore most valuable, and is probably not sufficiently appreciated by the public. It is certainly most arduous. It is hard for those who take up such work to find suitable work for their *protégés*. Apart from the natural disinclination of employers to take in a criminal, and the equally natural objection of workmen to have a "gaol-bird" as one of their mates, there is the difficulty of finding work within the capacity of a person whose early training has too often taught him little of the habit of industry, and who is fit for but few of the industries of modern life. When we consider their limitations, their temptations, the wonder is not that so many criminals relapse into their old ways, but that any are redeemed. It can be done only by individual effort directed to individual cases. The personal element is the mainspring of success. It is therefore a work that must be largely left to private benevolence; it cannot be done in wholesale fashion. But the fact that it cannot be done by the State should not make us ignore that it is in every way a national benefit.

